# A Case of Suspected Neurosarcoidosis Evading Diagnosis With Cervical Biopsy

**DOI:** 10.7759/cureus.62540

**Published:** 2024-06-17

**Authors:** Noah King, Madhu Vishnu Sankar Reddy Rami Reddy, Andrew Waack, Alastair Hoyt, Jason Schroeder

**Affiliations:** 1 Neurosurgery, The University of Toledo College of Medicine and Life Sciences, Toledo, USA

**Keywords:** cervical biopsy, myelopathy, epidural abcess, cervical kyphosis, uncertain pathology, neurosarcoidosis

## Abstract

Neurosarcoidosis is a rare manifestation of sarcoidosis, posing diagnostic challenges due to its varied clinical presentation and the lack of definitive diagnostic tests. We present a case of a 46-year-old African American female with progressive ascending bilateral sensory loss, weakness, and a bifrontal headache. Despite undergoing extensive diagnostic workup including cerebrospinal fluid analysis, neuroimaging, and bronchoscopic evaluation, a definitive diagnosis remained elusive. The patient underwent an open cervical spinal cord biopsy, which did not yield conclusive evidence of neurosarcoidosis. Subsequent complications included suspicion of an epidural abscess and post-operative cervical kyphosis. This case underscores the diagnostic dilemma and potential complications associated with the evaluation and management of neurosarcoidosis, highlighting the importance of a multidisciplinary approach in such cases.

## Introduction

Sarcoidosis is an auto-inflammatory granulomatous disorder typically characterized by lymphatic and lung involvement, with variable involvement of multiple organ systems [1]. Current literature estimates that 5% of patients with sarcoidosis have nervous system involvement [2]. Most commonly, patients present in their early 40s with symptoms of cranial neuropathy; however, presentation can vary widely [2-4]. Additionally, there is no evidence of systemic disease in approximately 10-17% of patients with neurosarcoidosis [3]. Systemic disease often provides a nidus for biopsy outside the nervous system, aiding in diagnosis [5]. The degree of varying clinical presentation, differential etiologies, and the eloquence of the nervous system make diagnosis of neurosarcoidosis challenging.

In cases with neurological signs, without evidence of systemic disease, the cervical cord biopsy emerges as a valuable tool for facilitating directed biopsy of suspected sites of pathology [6]. Biopsy can present evidence of granulomatous inflammation, a key indicator of sarcoidosis [7]. Executing cervical cord biopsy involves meticulous technique, ensuring precision while minimizing procedural risks. Patients are positioned prone under general anesthesia, with the cervical biopsy site localized using fluoroscopy or ultrasound imaging. Following a posterior midline incision, exposure is created along with a laminectomy to visualize the cervical spine. Specialized biopsy needles or forceps are used to extract representative tissue samples. Continuous monitoring of neurological function is imperative to promptly identify and address any potential deficits [8]. While serving as a diagnostic asset, open cervical cord biopsy inherently carries risks of infection, neurological deficits, blood loss, and scar tissue formation [8]. These risks underscore the importance of clinicians conducting a thorough risk-benefit assessment before deciding to proceed with cervical cord biopsy, weighing the necessity for a definitive diagnosis against potential adverse outcomes [8].

## Case presentation

**Figure 1:**
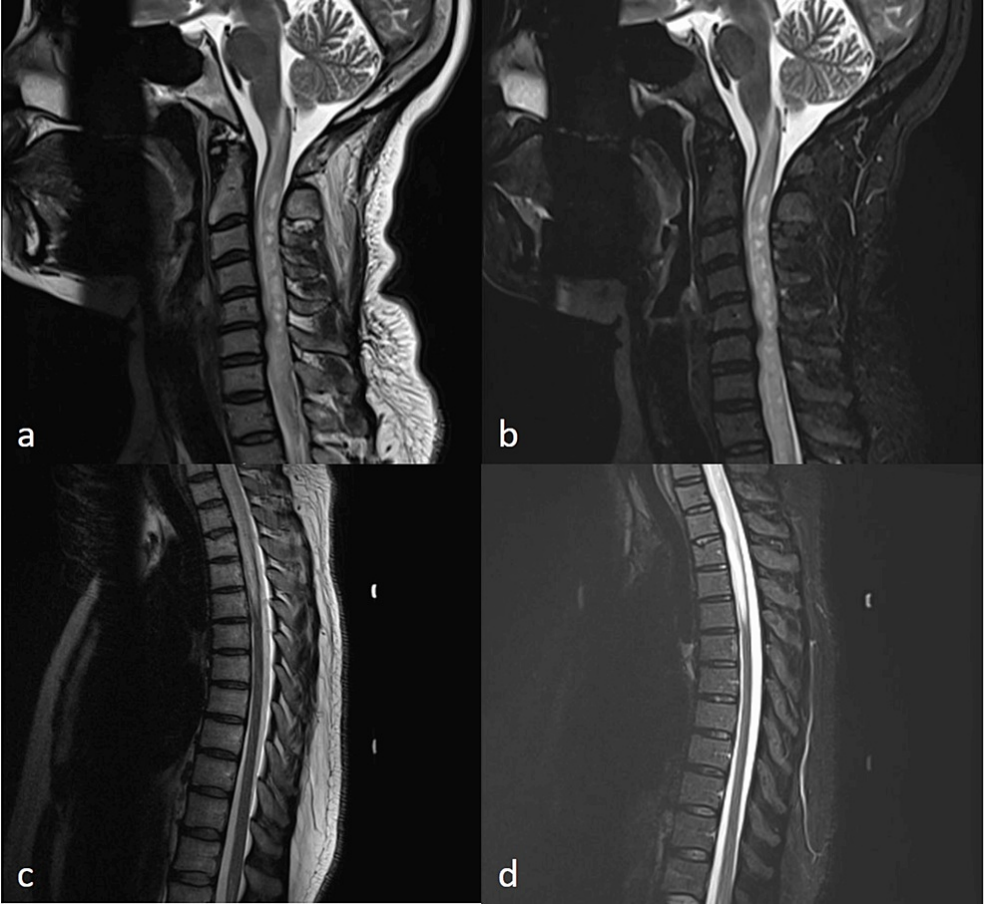
Sagittal T2 (a and c) and T2 short-tau inversion recovery (b and d) weighted magnetic resonance images of the cervical (a and b) and thoracic (c and d) spine. Cervical images show extensive cervical spinal cord signal abnormality with thick enhancement of the posterior aspect of the spinal cord and/or adjacent dura. At C5-6, there is degenerative disc disease with disc bulge spurs and cord flattening. At C6-7, there is degenerative disc disease with mild disc bulge and mild cord flattening. Thoracic images show diffuse signal abnormality in the upper thoracic cord to the T5-6 level with thick posterior enhancement to the T2-3 level.

**Figure 2:**
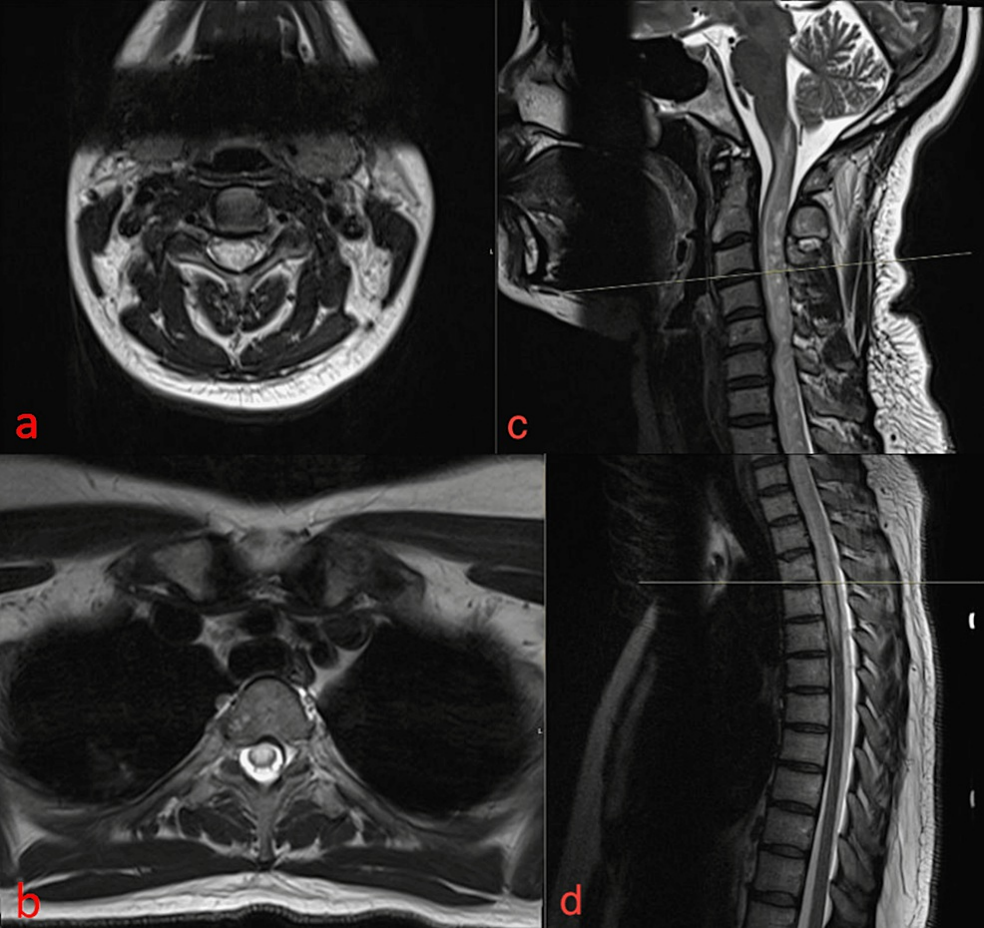
Axial localizer T2 weighted magnetic resonance images of the cervical (a) and thoracic (b) spine. Sagittal T2 (c and d) weighted magnetic resonance images of the cervical (c) and thoracic (d) spine. Axial images (a and b) show similar findings to sagittal (c and d) images, which are also included in Figure 1, with signal abnormality with enhancement of the posterior aspect of the spinal cord/adjacent dura.

The patient is a 46-year-old African American female with no significant past medical history presenting to the emergency department with a one-month history of progressive ascending bilateral sensory loss and weakness in the lower limbs, with recent symptom onset in the upper extremities. Symptoms were initially transient but progressed to constant numbness from the toes to the inferior border of the rib cage within the week before presentation. She reported associated balance difficulty, dropping items, bladder incontinence, and a bifrontal dull headache. Due to the progression of symptoms that impaired daily living, she was admitted for inpatient care. On admission, neurological examination revealed predominately impaired vibratory, proprioceptive, and fine-touch sensation in all extremities, with sensory ataxia and positive Romberg sign. Initial computed tomographic (CT) imaging of the brain and spine was unremarkable; however, reticulonodular opacities in the right lobe were incompletely identified on thoracic spine CT views. Cervical and thoracic MRI revealed posterior contrast enhancement of the dorsal column of the spinal cord (Figures 1, 2). The initial differential diagnoses primarily included, but were not limited to, subacute combined degeneration due to vitamin B12, vitamin E or copper deficiency, transverse myelitis, sarcoidosis, or a lung paraneoplastic phenomenon. Initial management included vitamin B12 supplementation and methylprednisolone 500 mg twice daily. 

**Figure 3:**
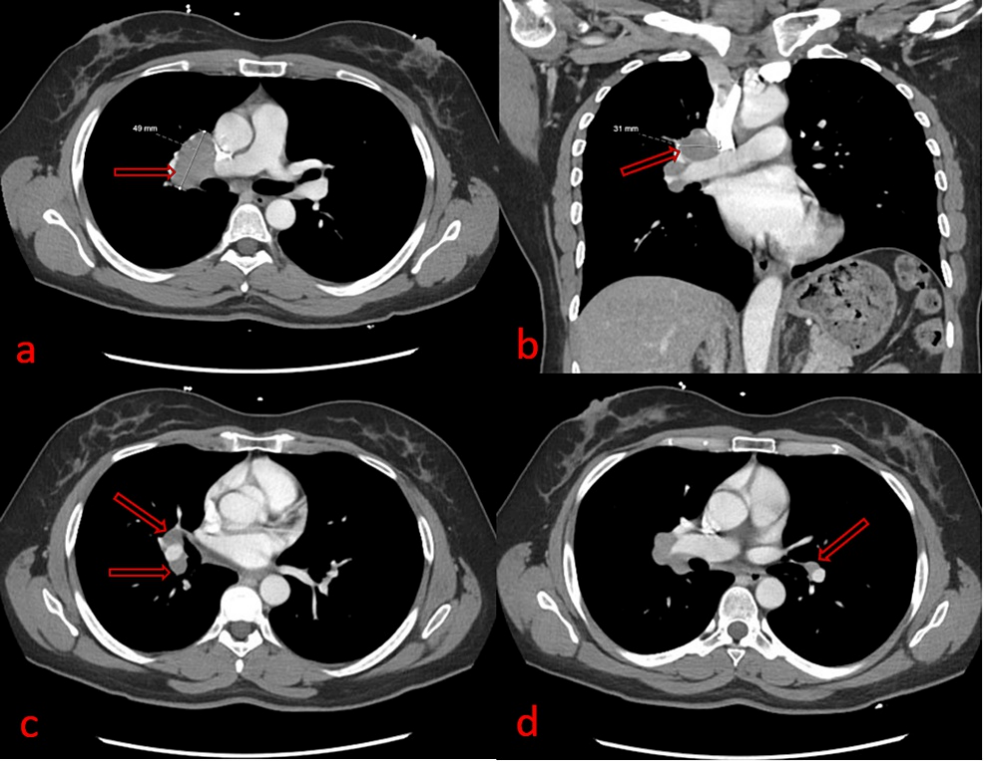
Chest axial (a, c, and d) and coronal (b) computed tomography images (CT) with contrast showing multiple enlarged right hilar lymph nodes (a, b, and c) and mildly enlarged left hilar lymph node (d). The largest enlarged right hilar lymph node measures 4.9 x 3.1 cm (a and b).

Further evaluation of the lung nodules included a CT of the chest with contrast (Figure 3), revealing multiple enlarged right hilar lymph nodes, a 2 mm indeterminate pulmonary nodule of the right lower lobe, and tree-in-bud nodular opacities in the right upper lobe. Indeterminate low-attenuation hepatic lesions were also identified; however, further abdominal MRI revealed the offending lesions to be consistent with hepatic cysts. A lumbar puncture was obtained which revealed the following: nucleated cells 8 cells/μL, lymphocytes 96%, protein 106 mg/dL, glucose 60 mg/dL, IgG cerebral spinal fluid (CSF): 13 mg/dL (elevated), IgG/albumin ratio: 0.2 (elevated), central nervous system (CNS) IgG synthesis: 5 mg/day (elevated). Cytology showed no malignant cells. Oligoclonal bands, neuromyelitis optica (NMO)/aquaporin-4 (AQP4), and myelin oligodendrocyte glycoprotein (MOG) were negative. Encephalitis and paraneoplastic panel (ENC2) were negative for all antibodies tested.

After five days of methylprednisolone treatment, the patient experienced a resolution of tingling paresthesia in all four extremities. The lack of a definitive diagnosis at this time prompted bronchoscopy with endobronchial ultrasound biopsy of the right hilar and carinal lymph nodes, in addition to bronchoalveolar lavage. Results showed no malignant cells or granulomas; all three biopsied nodes were significant for the mixed lymphoid population. Immunophenotypic analysis of the lymphoid cells demonstrated a mixed population of phenotypically unremarkable T-cells and polyclonal B-cells with no monoclonal lymphoid population detected. Bronchoalveolar lavage showed no malignant cells. She was discharged with prednisone 40 mg daily and was scheduled for an outpatient follow-up. A week later, she was evaluated by the neurosurgery team for a dural biopsy due to worsening symptoms with concern for neurosarcoidosis. She consented to multi-level laminectomy and biopsy of cervical dura, arachnoid, and posterior columns the next day. 

She underwent posterior laminectomy of C3-C4 to expose the thecal sac. Upon inspection, no clear abnormality was visualized in the epidural space. The dura was opened and tacked laterally, exposing the posterior cervical spinal cord. The arachnoid mater appeared cloudy and was harvested via microscissors for final pathology. The dorsal columns were identified and appeared anatomically normal with slight enlargement. The arachnoid knife was used to free tissue and the micro pituitary rongeur to harvest two small samples of the right dorsal column for final pathology. Before dura closure, a small dural sample of the medial edge was also obtained for pathological analysis. 

Preliminary pathology findings at the local institution indicated chronic inflammation of spinal cord tissue, benign arachnoid tissue with a focal cyst-like area, and benign dura. Immunostains revealed a few CD20-positive cells and focal CD68-positive cells, but no granulomas were identified. Final pathology results from an outside institution, analyzing the leptomeninges/dura and parenchyma, spinal cord biopsy indicated: “Spinal cord parenchyma with perivascular lymphocytic cuffs, composed of mixed chronic lymphocytes and reactive microglia/macrophages”. No definitive evidence of neurosarcoidosis was obtained. 

**Figure 4:**
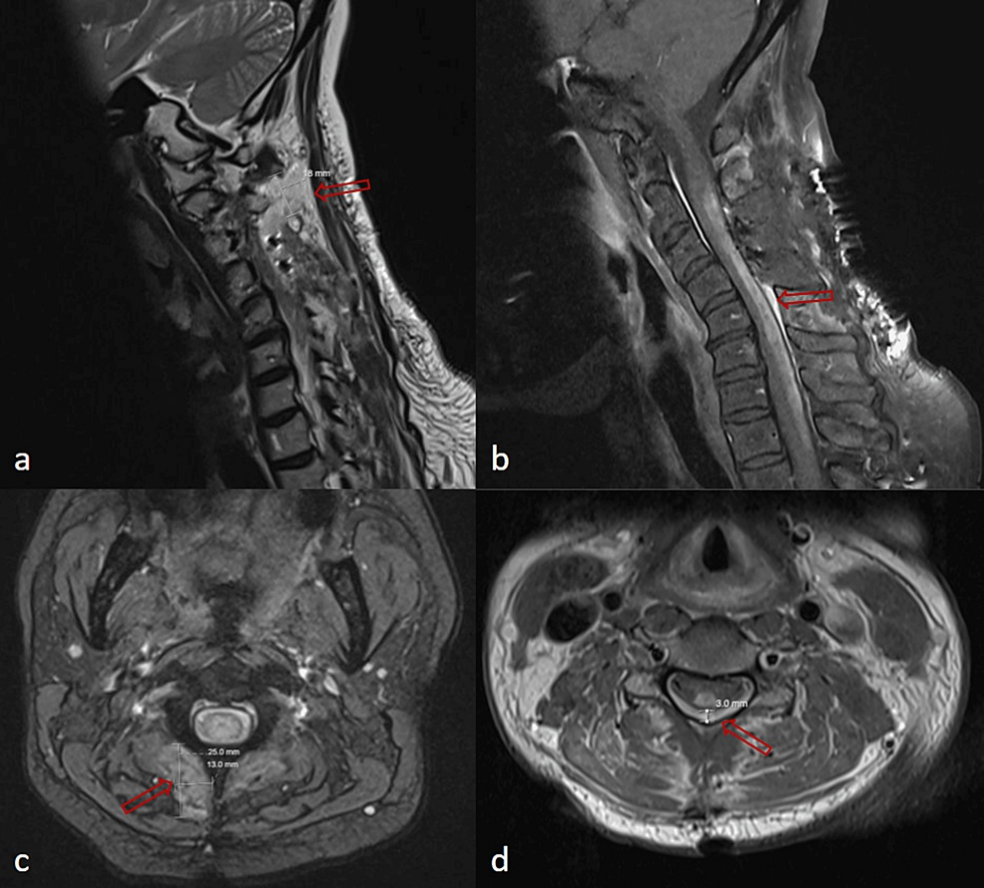
Sagittal (a and b) and axial (c and d) T2 (a and c) and T1 fat-saturated (b and d) weighted magnetic resonance images. Extensive postoperative edema and enhancement throughout the soft tissues including a 2.5 x 1.3 x 1.8 cm collection at the right paraspinal musculature of C2-4. (a and c, red arrow). Additionally, there is a hyperintense, enhancing, dorsal epidural collection measuring up to 3 mm in AP dimension and extends from C4-T1 (b and d, red arrow), possibly reflecting an epidural abscess.

One week postoperatively, the patient experienced worsening left arm and leg weakness. Repeat cervical MRI revealed a paraspinal fluid collection and a 3 mm epidural fluid collection from C4-T1, associated with enhancement compatible with epidural abscess (Figure 4). The patient consented to a washout procedure the next day for infection precautions. Another procedure was performed, reopening the surgical site at C3-C4 to access the epidural space. Epidural fluid and residual ligamentum flavum were taken for culture. No significant abscess or infection was found intraoperatively. A surgical drain was placed and maintained for three days post-operatively for fluid drainage. Four days later, culture revealed coagulase-negative staphylococcus and the patient started a six-week course of vancomycin. Due to the low virulence of the pathogen, corticosteroids were continued to address the underlying myelopathy. 

**Figure 5:**
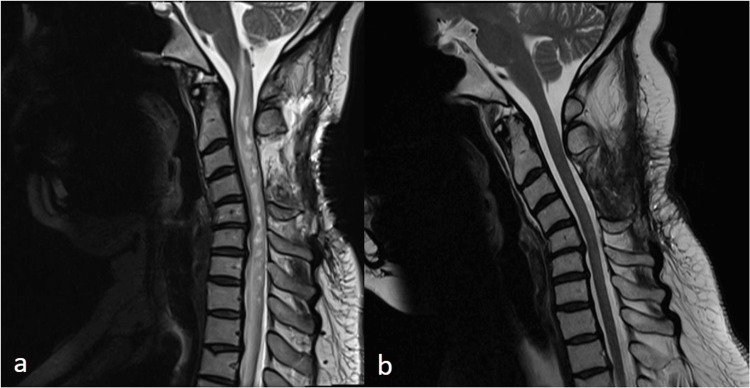
Sagittal T2 weighted magnetic resonance images of the cervical spine at one month post-operatively (a) and six months post-operatively (b). Images at one month demonstrate worsening enhancement within the dorsal cord, new enhancement within the operating site and cervical kyphosis. At six months there is no residual abnormal signal or pathologic contrast enhancement within the dorsal cord to suggest progressive or recurrent spinal cord disease.

Upon inconclusive biopsy findings, the patient was managed by the neurology team for inflammatory myelitis. She was treated with prednisone 120 mg daily, and any attempts to taper the steroids resulted in the exacerbation of myelopathy symptoms. Repeat cervical MRI was obtained one month post-operatively (Figure 5A). She was started on rituximab 1000 mg IV (every six months) beginning three months post-operatively, with plans to taper steroids by 10mg/month. At her most recent follow-up, six months post-operatively, a repeat cervical MRI was obtained (Figure 5B) with resolution of the enhancing posterior cervical cord lesion. However, she reported continual difficulty with balance, gripping items, and persistent but non-progressing sensory deficits in her lower extremities and hands. The patient was referred to a neuroimmunologist for further care, as a primary pathological diagnosis had not been established. 

## Discussion

We present a complex patient in which the pathological diagnosis for neurological symptoms and findings remained elusive. With a presentation of myelopathic symptoms, posterior dorsal cord enhancement, and enlarged hilar lymph nodes, the primary suspicion was neurosarcoidosis. Notably, myelopathic symptoms occur in 5-26% of patients with neurosarcoidosis [9]. These symptoms warrant a workup to rule out numerous causes of myelitis, including nutritional, neurodegenerative, and vascular etiologies [9-11]. With the lack of systemic pathological evidence of non-caseating granulomas, a thorough diagnosis of exclusion must be made [1,9] In our case, despite a proper medical workup, evidence for other possible etiologies was not found, prompting further investigation. Open cervical spinal cord biopsy is a relatively uncommon procedure with little published literature regarding safety and efficacy. However, a general open biopsy can be accurate in as high as 98% [12]. In our case, open cervical cord biopsy did not result in definitive evidence of granulomatous inflammation. While the patient did not have any substantial procedural neurological deficits, a follow-up cervical MRI revealed new cervical kyphosis and possible epidural abscess/osteomyelitis. Kyphosis post-laminectomy is a known complication with incidence widely varying based on age, pre-operative alignment, and operative changes including facet resection and fusion [13]. Reported surgical site infection incidence after spine surgery varies widely from 0-18% [14]. Procedures with instrumented fusion have a higher incidence than simple decompression. Additionally, a posterior cervical approach is associated with a higher incidence of infections compared to an anterior cervical or posterior lumbar approach [15]. Furthermore, in our case, the patient had been on multiple courses of corticosteroids for approximately one month pre-operatively, which may have influenced post-operative infection risk. Therefore, weighing the benefits to risks is essential when electing an open cervical cord biopsy.

An alternative method of obtaining cervical biopsy tissue includes percutaneous CT-guided methods. Similar to open biopsy, there is currently little published evidence regarding its efficacy and safety; however, it is primarily used for obtaining cultures and diagnosing metastasis [16]. Percutaneous methods can be advantageous with decreased invasiveness, risk of infection, and need for anesthesia, though adequate tissue localization and quantity are a greater concern. In a review of 430 CT-guided spinal biopsies, the biopsy of the cervical tract had the lowest diagnostic rate (70%) when compared to thoracic, lumbar, and sacral tracts [17]. Furthermore, only core needle biopsies (>1.5 cm diameter) preserve tissue structure, while fine needle aspiration (1 mm) is limited to cytological study [17]. In our patient's case, while interventional radiology-guided percutaneous biopsy could have been utilized, a non-diagnostic biopsy would have left uncertainties about a true negative outcome. Nevertheless, completion of a successful diagnostic percutaneous biopsy could circumvent the increased risk of infection and reoperation associated with an open procedure. Further literature regarding the best methods for cervical spinal cord biopsy is necessary to guide future clinical decisions.

## Conclusions

In patients experiencing complex neurological disease, establishing a diagnosis of uncommon pathology can be exceptionally challenging. We present a complex neurological patient with strong clinical suspicion of neurosarcoidosis but no confirmed evidence of systemic disease. Due to the worsening disease, the patient and clinicians opted for an open cervical biopsy, an uncommon procedure that did not establish a definitive diagnosis. The patient underwent reoperation due to suspicion of an epidural abscess with further complications of post-operative cervical kyphosis. While arguably providing a less definitive answer, complications could have been avoided with a percutaneous CT-guided approach. More evidence evaluating open versus percutaneous biopsy in the cervical spine is needed to guide future diagnostic evaluations.
